# PD-L1 expression and microsatellite instability (MSI) in cancer of unknown primary site

**DOI:** 10.1007/s10147-024-02494-3

**Published:** 2024-03-25

**Authors:** João Neif Antonio Junior, Daniel D.’Almeida Preto, Maria Eduarda Zanatta Neder Lazarini, Marcos Alves de Lima, Murilo Bonatelli, Gustavo Noriz Berardinelli, Vinicius Duval da Silva, Céline Pinheiro, Rui Manuel Reis, Flavio Mavignier Cárcano

**Affiliations:** 1 Department of Clinical Oncology, Barretos Cancer Hospital, Barretos, Brazil; 2 Molecular Oncology Research Center, Barretos Cancer Hospital, Barretos, Brazil; 3 Barretos School of Health Sciences Dr. Paulo Prata – FACISB, Barretos, Brazil; 4 Medical School, Life and Health Sciences Research Institute (ICVS), University of Minho, Braga, Portugal; 5 3ICVS/3B’s-PT Government Associate Laboratory, Braga, Portugal; 6 Oncoclinicas & Co - Medica Scientia Innovation Research (MEDSIR), Sao Paulo, Brazil

**Keywords:** Neoplasms, Unknown primary, Microsatellite instability, Immune checkpoint proteins, Immunotherapy

## Abstract

**Background:**

Cancer of unknown primary site (CUP) is a heterogeneous group of tumors for which the origin remains unknown. Clinical outcomes might be influenced by regulatory processes in its microenvironment. Microsatellite instability (MSI) is a predictive biomarker for cancer immunotherapy and its status, as well as co-occurrence with PD-L1 expression, is poorly evaluated. We aim to evaluate the expression of PD-L1 and the status of MSI in CUP and their possible associations with clinical–pathological features.

**Methods:**

The combined positive score (CPS) PD-L1 expression was evaluated by immunohistochemistry. MSI status was assessed using a hexa-plex marker panel by polymerase chain reaction followed by fragment analysis.

**Results:**

Among the 166 cases, MSI analysis was conclusive in 120, with two cases being MSI positive (1.6%). PD-L1 expression was positive in 18.3% of 109 feasible cases. PD-L1 expression was significantly associated with non-visceral metastasis and a dominance of nodal metastasis. The median overall survival (mOS) was 3.7 (95% CI 1.6–5.8) months and patients who expressed PD-L1 achieved a better mOS compared to those who did not express PD-L1 (18.7 versus 3.0 months, *p*-value: < .001). ECOG-PS equal to or more than two and PD-L1 expression were independent prognostic factors in multivariate analysis (2.37 and 0.42, respectively).

**Conclusion:**

PD-L1 is expressed in a subset (1/5) of patients with CUP and associated with improved overall survival, while MSI is a rare event. There is a need to explore better the tumor microenvironment as well as the role of immunotherapy to change such a bad clinical outcome.

## Introduction

Cancer of unknown primary site (CUP) is a group of malignant neoplasms diagnosed by biopsy of metastasis, but with no primary cancer identified after a comprehensive clinical and radiological assessment [1, 2]. They account for 3% up to 5% of the most common tumors in developed countries, and the average diagnosis is at 60 years old, with a similar incidence between men and women [2]. Five histological subtypes are frequently identified, and the large majority are adenocarcinomas, mainly well or moderately differentiated [2]. Immunohistochemistry is a fundamental part of the diagnosis workup, yet it can fail to define the precise origin of the tumor [3, 4]. Moreover, even new image resources such as FDG-PET/CT (Fluorodeoxyglucose positron emission tomography computed tomography) lack better sensitivity and specificity to detect the primary site [5].

Chromosome abnormalities and overexpression of genes such as *EGFR*, *Kit/PDGFR*, *RAS*, *BCL2*, and *ERBB2* are described in CUPs [6, 7]. CUPs have substantial mutational heterogeneity. *TP53, MUC16, KRAS, LRP1B and CSMD3* are the most frequently mutated genes, along with *FGFR2* being the most common gene involved in fusion events [8]. In the last decade, genomic profiling has been assessed to define the histology of the primary site, and druggable molecular targets have been described [9–11]. There are several molecular signatures to predict the tissue of origin in CUPs with variable accuracy, but yet some uncertainties of their benefits exist in routine clinical use [12, 13].

A wide variety of clinical presentations might be noted in patients with CUPs, and their biological behavior is commonly aggressive and somehow unpredictable, leading to a worse prognosis [14]. There is no standard treatment regimen, but some benefits in response rate and survival can be observed from taxane- or platin-based chemotherapy [15, 16].

The immune checkpoint molecule programmed death-1 (PD-1) plays a role in self–nonself discrimination by the immune system [17]. PD-1 expression is high on T cells in the tumor microenvironment, and its primary ligand, PD-L1, is variably expressed on tumor cells and antigen-presenting cells within tumors, providing a potent inhibitory effect within the tumor microenvironment [18]. In the last decade, immune checkpoint inhibitors, such as anti-PD-1 or anti-PD-L1 drugs, have been largely explored as a therapeutic strategy in oncology [19, 20]. Microsatellite instability (MSI) is a marker of genetic instability, mainly due to epigenetic or genetic silencing of mismatch repair pathway genes [21]. Based on the significative overall response rate, FDA approved pembrolizumab (anti-PD-1) to treat patients, in an agnostic way, whose solid tumor harbors mismatch repair deficiency [22]. The PD-L1 expression and the presence of MSI in the CUP microenvironment have been underexplored.

Herein, we aim to explore the expression of PD-L1 and the frequency of MSI in a representative cohort of CUP.

## Patients and methods

### Study population and selection of cases

We identified 166 cases of CUP patients who presented to Barretos Cancer Hospital between 2002 and 2016. Clinical–pathological features were retrieved from the medical records and collected using REDCap electronic data capture tool [23]. All included patients underwent immunohistochemistry in accordance with appropriate diagnostic guidelines and immunohistochemical analysis and the results failed to define the primary site [24]. All men underwent thorough image tests with no diagnosis of primary site. All patients included had been tested for prostate-specific antigen (PSA) and human chorionic gonadotropin (HCG) in cases of undifferentiated carcinoma. We excluded patients with exclusive head and neck squamous cell carcinoma in the lymph nodes, women with exclusive axillary adenocarcinoma in the lymph nodes or peritoneum involvement of adenocarcinoma, and midline tumors in young adults. Histology such as neuroendocrine tumors, melanoma, and small-cell tumors were excluded, as well as patients with chronic immunosuppression history.

This study was approved by the local IRB under Protocol No. 1055/2015.

### PD-L1 Immunohistochemistry

The immunohistochemistry reaction was performed using BenchMark Ventana Ultra™ (Ventana, Tucson, AZ, USA) platform, through multimer linked to horse radish peroxidase, to detect PD-L1 protein, as previously reported [25]. The anti-PD-L1 (E1L3N®) XP® Rabbit mAb, Cell Signaling Technology, was used as primary antibody and we used the OptiView DAB IHC Detection Kit, following manufacturer’s guidelines. Placental syncytiotrophoblast was used as positive control tissue. The combined positive score (CPS) was used to measure the expression of PD-L1. CPS corresponds to the ratio between the total of PD-L1 positive cells (tumor cell, lymphocytes and macrophages) and the total of viable tumor cells, multiplied by 100 [26]. Considering the experience of appropriateness of CPS cutoff in other types of tumors and no agreement of CPS cutoff for CUPs, we used CPS ≥ 1 in our study [27].

### DNA isolation and microsatellite instability (MSI) assay

Tumor DNA was isolated from FFPE sections using QIAamp DNA Micro Kit (Qiagen, Germany), as previously reported [28]. Briefly, the MSI assay was performed using HT-MSI + kit (Cellco, São Carlos, Brazil) composed of six quasi-monomorphic mononucleotide repeat markers (NR27, NR21, NR24, BAT25, BAT26, and HSP110), following the manufacturer’s guideline. The analysis was performed using 3500 Genetic Analyzer automated sequencer (Applied Biosystems, USA) and analyzed by the GeneMapper software (Applied Biosystems, USA), according to manufacturer's recommendations. Cases with the presence of two or more markers out of the quasimonomorphic variation range (QMVR) were classified as MSI positive (MSI +), and cases without markers out of QMVR were classified as MSI negative (MSI −).

### Statistical analysis

Univariate analysis was used to associate sample variables in relation to PD-L1 expression, using the χ2 test or Fisher’s exact test, according to the characteristics of the sample. Survival curves were plotted using the Kaplan–Meier method and the event of interest (death) was considered for the outcome of overall survival (OS). Alive patients and those lost to follow-up were censored. Univariate and multivariate analysis were performed using Cox regression method. Statistical analyses were performed using IBM SPSS Statistics for Mac OS, Version 2.0 (IBM). *P* values < 0.05 were considered statistically significant.

## Results

### Clinical–pathological characterization of CUP patients

One hundred and sixty-six cases of CUPs were identified according to selection criteria (Table 1). The mean age was around 60 years, and gender, tobacco exposure, and cancer family history did not correlate with PDL1 status. Most patients were non-alcohol drinkers and were diagnosed with adenocarcinoma, lymph node metastasis, visceral metastasis as a dominant site, and good performance status. Less than half of the patients received first-line chemotherapy and the majority were treated with carboplatin/paclitaxel combination. During the pathological assessment by immunohistochemistry, 33 different tissue biomarkers were used considering the whole series, and CK7, CK20, TTF-1, vimentin, and CEA were the most common (93.5%, 88.0%, 78.7%, 71.3% and 65.7%, respectively). Serum biomarkers such as CEA, CA 19.9, CA 15.3, or alpha-fetoprotein were requested in 49.4% of cases and at least one of them was altered in 78% of those cases.

**Table 1:** Clinicopathological features of the CUP patients

Variable	*n* (%)
CUPs patients	166
Age (years)	
Mean [SD]	60.4 [12.4]
Min–max	20–89
Gender	
Male	83 (50.0)
Female	83 (50.0)
Smoking	
Current or former smoker	73 (44.0)
Non-smoker	77 (46.4)
Unknown	9.6 (16.0)
Alcohol consume	
Current or former alcohol drinker	40 (24.1)
Non-alcohol drinker	104 (62.7)
Unknown	22 (13.3)
Cancer family history	
Yes	64 (38.6)
No	72 (43.4)
Unknown	30 (18.1)
Histology	
Adenocarcinoma	104 (62.6)
Carcinoma	43 (26.0)
Squamous cell carcinoma	19 (11.4)
Metastasis site	
Lymph node	99 (59.6)
Liver	83 (50.0)
Lung	54 (32.5)
Bone	68 (41.0)
CNS	9 (5.4)
Other	34 (20.5)
Biopsy site (tissue analyzed)	
Liver	35 (32.1)
Lymph node	32 (29.4)
Bone	26 (23.9)
CNS	4 (3.7)
Other	12 (11.0)
Dominant site of metastasis	
Visceral	76 (45.8)
Lymph node	41 (24.7)
Bone	31 (18.7)
CNS	5 (3.0)
Other	13 (7.8)
Performance Status ECOG	
< 2	63 (38.0)
≥ 2	56 (33.7)
Unknown	47 (28.3)
Treatment cathegory	
Surgery any time	21 (12.7)
Radiation therapy any time	52 (31.3)
1st Line chemotherapy	80 (48.2)
2nd Line chemotherapy	28 (16.9)
> 2 Lines of chemotherapy	11 (6.6)
1st Line type of chemothterapy	
Carboplatin/Paclitaxel	42 (25.3)
Cisplatin/Gemcitabine	9 (5.4)
Other	29 (17.5)
Not applicable	86 (51.8)

SD: Standard deviation; CNS: Central Nervous System

### PD-L1 expression and outcomes

**Fig. 1:**
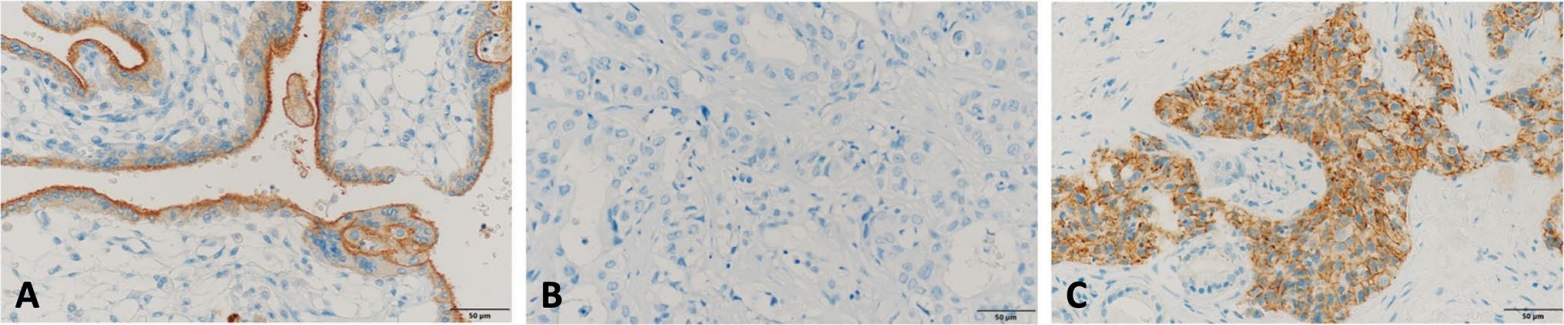
Micrographs of immunohistochemistry staining and the expression of PD-L1 in CUP tumor cells (400x). From the left to the right: **A** PD-L1 positive control; **B** negative PD-L1 expression in liver metastasis of carcinoma; **C** positive PD-L1 expression in lymph node metastasis of carcinoma

Fifty-seven cases were excluded from immunohistochemistry analysis, due to technical issues or unavailable biological material. A total of 109 cases were analyzed for PD-L1 expression. Using the CPS score, it was found that 18.3% expressed PD-L1 (Fig. 1).

PD-L1 expression was significantly associated with non-visceral metastasis and a dominance of nodal metastasis, as well as biopsies from lymph nodes (Table 2).

**Table 2:** Association between PD-L1 staining and clinicopathological features in CUP patients

Clinical features	*n*	PD-L1 staining*		*p-value*
		Negative (%)	Positive (%)	
Evaluable CUPs	109	89 (81.7)	20 (18.3)	
Age (years)				
< 60.4	53	40 (44.9)	13 (65.0)	0.100
≥ 60.4	56	49 (55.1)	7 (35.0)	
Gender				
Male	48	41 (46.1)	7 (35.0)	0.360
Female	61	48 (53.9)	13 (65.0)	
Histology				
Adenocarcinoma	65	52 (58.4)	13 (65.0)	0.840
Carcinoma	33	28 (31.5)	5 (25.0)	
Squamous cell carcinoma	11	9 (10.1)	2 (10.0)	
Visceral metastasis				
Yes	65	57 (64.0)	8 (40.0)	0.048
No	44	32 (36.0)	12 (60.0)	
Dominant site of metastasis				
Visceral	44	41 (46.1)	3 (15.0)	< 0.001
Lymph node	29	20 (22.5)	9 (45.0)	
Bone	25	23 (25.8)	2 (10.0)	
CNS	4	2 (2.2)	2 (10.0)	
Other	7	3 (3.4)	4 (20.0)	
Biopsy site (tissue analyzed)				
Liver	35	33 (37.1)	2 (10.0)	0.003
Lymph node	32	20 (22.5)	12 (60.0)	
Bone	26	23 (25.8)	3 (15.0)	
CNS	4	2 (2.2)	2 (10.0)	
Other	12	11 (12.4)	1 (5.0)	

CNS: Central Nervous System; CPS: Combined Positive Score; (*) according to CPS and cutoff of 1 (1 up to 80)

**Fig. 2:**
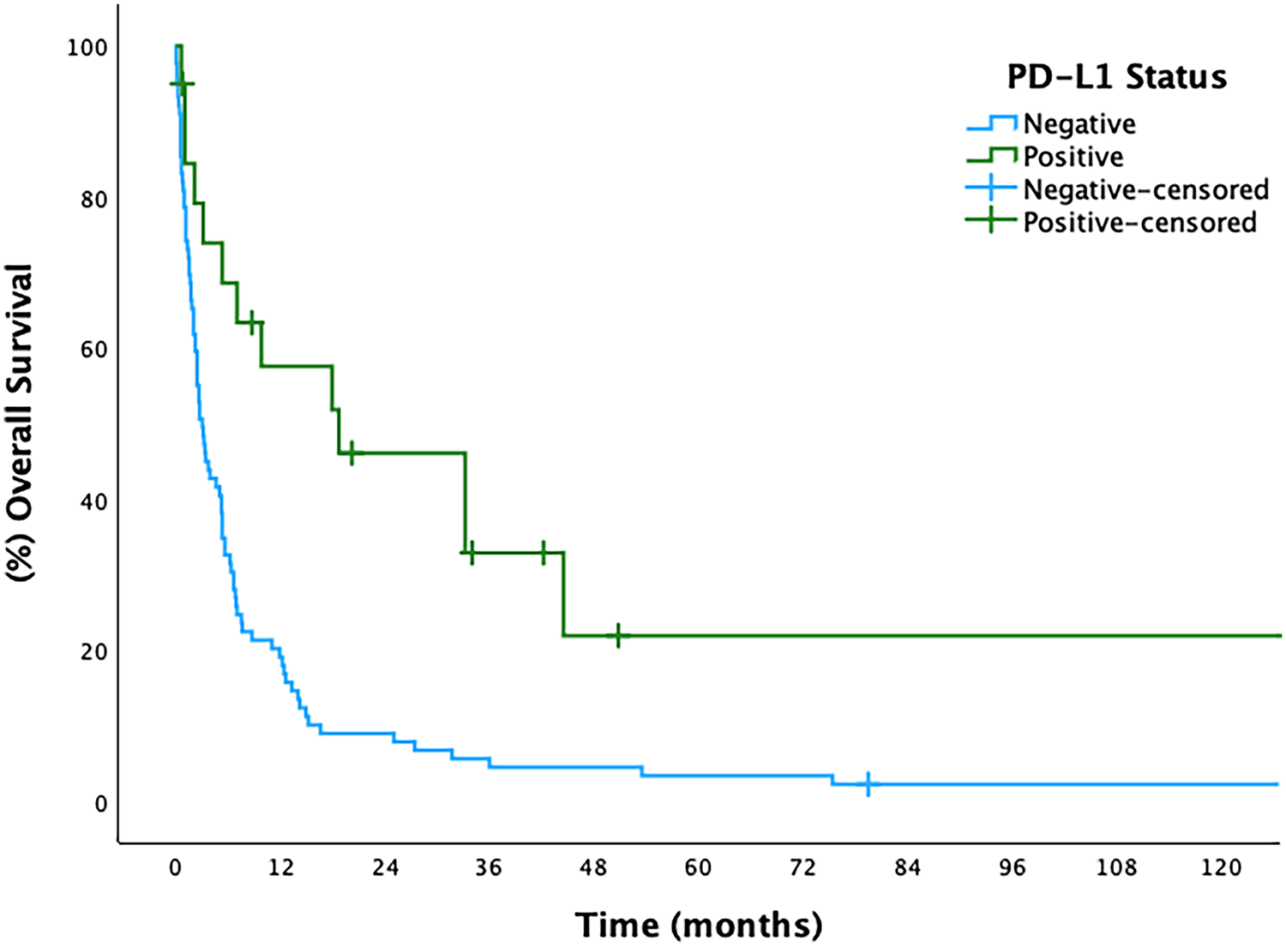
Kaplan–Meier overall survival curve according to PD-L1 expression by CPS

The median overall survival was only 3.7 (95% CI 1.6–5.8) months and patients who expressed PD-L1 obtained better median overall survival compared to those who did not express it (18.7 versus 3.0 months, *p*-value: < 0.001)(Fig. 2). The presence of visceral metastasis and PS ECOG equal to or more than two were associated with increased risk of death, while PD-L1 expression was a protective factor in univariate analysis (Table 3). However, in the multivariate analysis, only PS ECOG equal to or more than two and PD-L1 expression were independent prognostic factors (HR: 2.37 and 0.42, respectively) (Table 3).

**Table 3:** Univariate and multivariate Cox regression analysis of overall survival in CUP patients

Characteristics	Univariate analysis			Multivariate analysis		
	HR	95% CI	*P* value	HR	95% CI	*P* value
Age (years)						
< 60.4	Ref					
≥ 60.4	1.19	0.80–1.77	0.390			
Gender						
Female	Ref					
Male	1.12	0.75–1.67	0.560			
Histology						
Adenocarcinoma	Ref					
Carcinoma	0.86	0.55–1.35	0.510			
Squamous cell carcinoma	0.80	0.40–1.57	0.510			
Visceral metastasis						
No	Ref			Ref		
Yes	1.8	1.19–2.72	0.005	1.43	0.94–2.19	0.100
Dominant site of metastasis						
Other	Ref					
Bone	1.00	0.40–2.48	0.980			
Visceral	1.45	0.61–3.46	0.390			
Lymph node	0.68	0.27–1.67	0.400			
CNS	0.83	0.23–2.99	0.780			
ECOG Performance Status						
< 2	Ref			Ref		
≥ 2	2.65	1.67–4.20	< 0.001	2.37	1.50–3.76	< 0.001
Unknown	1.96	1.15–2.33	< 0.001	1.69	0.98–2.90	0.060
PD-L1 staining*						
Negative	Ref			Ref		
Positive	0.35	0.20–0.64	< 0.001	0.42	0.23–0.76	0.0050

OS: Overall survival; CNS: Central Nervous System; (*) according to Combined Positive Score and cutoff of 1 (1 up to 80)

### Microsatellite instability analysis

**Fig. 3:**
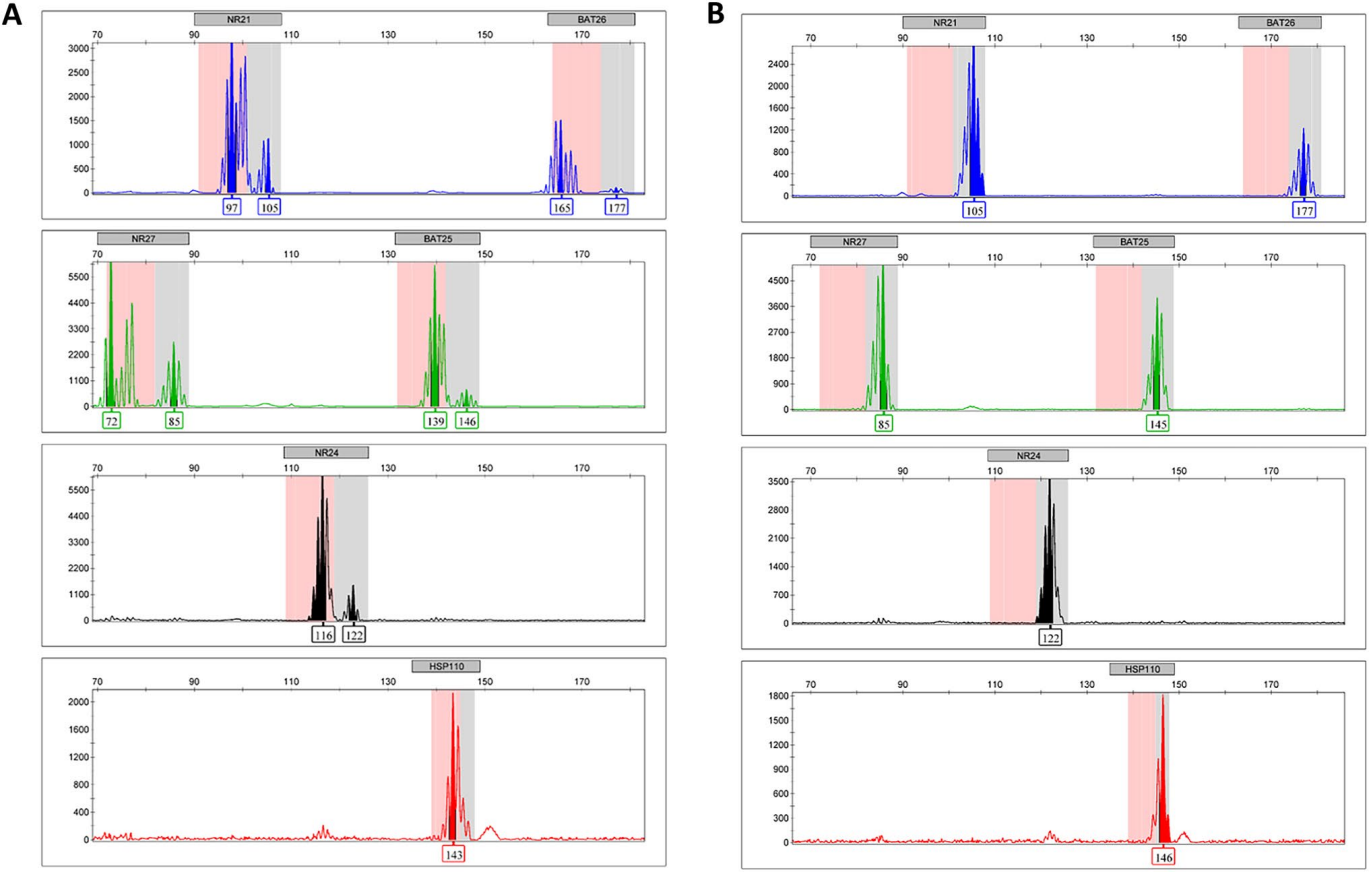
Microsatellite instability (MSI) analysis. **A** MSI-positive (MSI +) phenotype case. **B** MSI-negative (MSI-) phenotype case. X axis: Microsatellite Marker (base pair, bp). Y axis: Relative Fluorescence Unit (RFU)

MSI analysis was conclusive in 120 cases, with 46 being excluded due to insufficient/inappropriate DNA quality or biological material unavailability. We found that only 2 out of the 120 cases (1,6%) were MSI positive (Fig. 3). One patient was a 46-year-old woman with a family history of breast cancer, diagnosed with metastatic adenocarcinoma in lymph nodes, the sole site of metastasis. She received chemotherapy comprising a combination of carboplatin and paclitaxel, achieving 8 months of progression-free time. The patient received gemcitabine as second-line treatment with no response in 3 months and died 14 months after diagnosis. The tumor did not express PD-L1. The other MSI-positive patient was a 40-year-old woman, smoker, and alcohol drinker, with a family history of prostate cancer, colorectal cancer, and lung cancer. The patient was diagnosed with metastatic adenocarcinoma in the lymph nodes, the dominant site of metastasis, and had liver metastasis. The patient received chemotherapy combining carboplatin and paclitaxel and achieved only 6 months of progression-free time. She received doxorubicin as second-line treatment with limiting toxicity and obtained no response. She died 18 months after diagnosis. The tumor highly expressed PD-L1 with CPS of 25.

## Discussion

Despite the breakthroughs in cancer therapeutics in the last decades, CUP remains a difficult prognostic disease. In the present study, we analyzed a significant number of CUP cases, following strict selection criteria, to explore the role of PD-L1 and MSI.

Our cohort is in line with other reports that demonstrated a mean age around 60s and poor overall survival [29, 30]. In a large Canadian registry study, the median overall survival of CUP achieved only 2 months and was even poor for those that were unable to receive any treatment [31]. In our series, only half of the cases were able to receive first-line chemotherapy and the median overall survival was less than 4 months. None of the included patients received treatment with immune checkpoint inhibitors.

The PD-L1 expression has been well explored in tumors, but few studies have investigated it in CUPs. Haratani et al. showed that the expression of immune checkpoint biomarkers seems to be similar to that in other solid tumors that commonly respond to immune checkpoint inhibitors [32]. Twenty-eight percent of CUPs harbor some immune checkpoint biomarker such as MSI, PD-L1, or high tumor mutational burden, as well as around 22% of CUPs express PD-L1 using a 5% cutoff [33]. We found 18.3% of CUPs expressing PD-L1 using 1% cutoff with the E1L3N® immune assay antibody. Despite different cutoffs, immune assay antibodies and platforms (e.g., Dako, Roche Diagnostics, Ventana) have been used in different studies to assess PD-L1 expression [34, 35]. Koomen et al. addressed this issue in a systematic review and observed agreement among the different immune assays, including those that used E1L3N® immune assay antibody. [36] Although PD-L1 has been investigated in several types of tumors as a therapeutic predictive biomarker, even patients who do not express it might benefit from immune checkpoint inhibitors [37, 38].

We found a reduction of 58% on relative risk of death associated with PD-L1 expression in our study. At variance, some studies have associated PD-L1 expression to worse prognosis, putatively by promoting tumor immune evasion and consequent disease progression [39–41]. Other factors not yet evaluated in the tumor microenvironment might explain such outcome discrepancies. Huang et al. found that PD-L1 expression was associated with short survival in breast cancer. However, it is worth noting that PD-L1 expressed only in tumor infiltrated lymphocytes (TILs) and resulted in better survival, achieving a reduction of 59% on relative risk of death [42]. We did not investigate TILs in our study, and other studies have also reported different outcomes in different tumor types associated with PD-L1 expression [43, 44]. Different forms of extracellular PD-L1, such as on exosomes or as a freely soluble protein, have been described. Alongside other mechanisms, these might shed light on a better understanding of CUP microenvironment and clinical outcomes.

MSI is a genomic instability biomarker that is commonly associated with high tumor mutational burden [45]. Cancers with high frequency of MSI are likely to be immunogenic and led to FDA approving the immune checkpoint inhibitor pembrolizumab as an agnostic agent for all tumors harboring MSI [45]. We found only 1.8% of our cases harboring MSI, in line with frequencies reported by Gatalica et al. on 384 CUP cases [46]. Furthermore, the CUPISCO trial (NCT03498521) has been using comprehensive genomic profiling (CGP) to assign patients with CUP to targeted or immunotherapy treatment, and just 3 out of 96 cases were MSI positive (1%) [47]. Despite being rare, MSI in CUPs might open the opportunity to use immunotherapy, mainly in chemotherapy-refractory cases.

Our findings point toward immunotherapy as a potential therapeutic strategy for CUPs as is already seen in some early clinical trial results [48, 49]. Another aspect to point out is that over a half of the patients did not undergo chemotherapy, primarily due to their low clinical performance at diagnosis, highlighting the need for strategies aimed at earlier diagnosis and a shorter interval between diagnosis and the initiation of systemic treatment.

The present work has some limitations: it has a retrospective design and the number of patients was not large enough to allow statistical power, which may raise issues related to selection bias. Another issue is the single-institution nature, as issues concerning patient referral, treatment access, and service protocols might influence clinical outcomes, thus limiting the generalizability of the findings. Nevertheless, we employed a validated immunohistochemistry protocol, using strict selection criteria to maximize sample homogeneity and assembled a relevant case series, given the acknowledged rarity of the CUPs.

In conclusion, PD-L1 is expressed in a subset (circa 20%) of patients with cancer of unknown primary site and is an independent predictor of overall survival in a sample not exposed to immune checkpoint inhibitors. MSI is a rare event in these patients. The analysis of these two immune biomarkers can identify a group of CUP patients, who could benefit from immunotherapy approaches.
